# Application of ^18^F Prostate-Specific Membrane Antigen Positron Emission Tomography/Computed Tomography in Monitoring Gastric Metastasis and Cancer Thrombi from Renal Cell Carcinoma

**DOI:** 10.1155/2022/5681463

**Published:** 2022-02-04

**Authors:** Min Xiong, Weiguang Zhang, Chao Zhou, Junjie Bao, Shengbing Zang, Xiaoping Lin

**Affiliations:** ^1^Department of Nuclear Medicine, Sun Yat-Sen University Cancer Center, Guangzhou, China; ^2^State Key Laboratory of Oncology in South China, Collaborative Innovation Center for Cancer Medicine, Sun Yat-Sen University Cancer Center, Guangzhou, China; ^3^Department of Pathology, Sun Yat-Sen University Cancer Center, Guangzhou, China

## Abstract

**Background:**

Renal cell carcinoma (RCC) with gastric metastasis is rare, particularly accompanied by multiple cancer thrombi.

**Methods:**

We reported a 66-year-old man with a history of a right radical nephrectomy because of RCC. The patient underwent ^18^F prostate-specific membrane antigen (PSMA) positron emission tomography/computed tomography (PET/CT) scanning after 6 months of targeted therapy because of gastric metastasis and cancer thrombi. We conducted a systematic review of the literature and identified 73 cases of RCC with gastric metastasis. We analyzed the clinicopathological characteristics, therapies, and outcomes of patients.

**Results:**

^18^F-PSMA PET/CT showed a large mass in the gastric fundus and cancer thrombi in the right atrium, inferior vena cava, and splenic vein with intense tracer uptake. Other metastases with increased tracer uptake included multiple bones and abdominal lymph nodes. The majority of gastric metastasis of RCC were men (53/73, 72.6%), with a median age at presentation of 67 (from 48 to 87) years. Gastric metastasis of RCC was mainly metachronous, and presented with small polyps or mass appearance and often accompanied by multiple-site metastases and gastrointestinal symptoms. An overall median interval between nephrectomy and diagnosis of gastric metastasis was 6 (from 0.1 to 23) years, and an overall median survival time was 14 (from 0.25 to 72) months. The median interval time of solitary gastric metastasis was longer than gastric metastasis with multiple-site metastases (7 vs.5 years; *P*=0.034). Patients with gastric and multiple-site metastases had higher mortality than patients with solitary metastasis (17 vs.1; *P*=0.028). The patients with synchronous gastric metastasis had a shorter survival time than metachronous gastric metastasis (6 vs.17 months; *P*=0.018).

**Conclusions:**

Postoperative follow-up of multiple imaging modalities to monitor recurrence and metastasis is necessary and important. PSMA PET/CT can improve the detection sensitivity of RCC, especially in metastatic clear cell renal cell carcinoma (ccRCC), and could provide a basis for disease staging, restaging, and therapeutic efficacy evaluation.

## 1. Introduction

Renal cell carcinoma (RCC), a malignant neoplasm arising from the renal tubular epithelial system, is the most common solid lesion within the kidney. RCC usually presents a poor prognosis, and about 25% of patients are reported to be in an advanced stage of disease at diagnosis with local invasion or distant metastasis [1, 2]. Clinically, metastasis of RCC to the stomach and forming vascular cancer thrombi are rare. It is reported that cancer thrombi in the renal vein or inferior vena cava occur in about 5–10% of patients [3]. The probability of gastric metastasis is low, accounting for about 0.65% of RCC metastasis [4]. Therefore, there were few cases described in the literature and it is difficult to be recognized. Due to the lack of specific tumor markers, imaging is the main routine examination for follow-up after RCC treatment. Traditionally, computed tomography (CT) with the intravenous contrast agent has been the main imaging modality in RCC follow-up. However, it remains difficult to definitive small metastasis [5]. The prostate-specific membrane antigen (PSMA) is a type II transmembrane glycoprotein that was initially thought to be specific to the prostate but was subsequently found to be expressed in tumor-associated neovascularization in many cancer types, such as ccRCC [6, 7], glioma [8, 9], thyroid cancer [10], breast cancer [11], primary cholangiocarcinoma [12], and more. Consequently, it is a potential candidate for targeted imaging strategies. Preliminary studies involving the use of PSMA positron emission tomography/computed tomography (PET/CT) imaging in metastatic RCC have been encouraging with evidence of improved staging sensitivity and finding small lesions that have directly led to change in management in some cases [13–15].

Herein, we reported a patient with gastric metastasis, cancer thrombi, and multiple metastases to other organs after radical RCC surgery using ^18^F-PSMA PET/CT. Furthermore, we conducted a systematic review of the literature to report the collected cases of RCC patients with gastric metastasis.

## 2. Materials and Methods

### 2.1. Image Acquisition of ^18^F-PSMA PET/CT

For this patient, a standard scan protocol was performed in the department of nuclear medicine. Intravenous ^18^F-PSMA was injected (dose 140.6 MBq) and a whole-body ^18^F-PSMA PET/CT (uEXPLORER, United Imaging Healthcare, China) 3D tomographic acquisition was performed after resting for approximately 60 minutes. The PET image acquisition time was approximately 10 minutes for one bed. CT scan was performed with the PET scan for anatomic localization and attenuation correction.

### 2.2. Case and Literature Search

We collected and summarized the clinical and pathological data of a 66-year-old man with gastric metastasis from RCC, including imaging characteristics and treatments. We searched the literature (from 1964 to 2021) on this topic by a systematic PubMed search using the keywords “renal cell carcinoma,” “gastric metastasis” and “stomach metastasis.” The searched articles were used to identify relevant studies. We found 73 cases excluding those unrelated and repeated ones. Including our patient, total 74 patients who were diagnosed with gastric metastasis from RCC were reviewed. For each patient, we obtained data on age, gender, primary tumor type, data of gastric metastasis (location, size, number, and shape), other metastatic sites, examination modalities, the interval between nephrectomy of the primary tumor and the diagnosis of gastric metastasis, treatment methods, and outcomes. We analyzed these data to identify a possible relationship between clinical variables and survival.

### 2.3. Statistical Analysis

We used the *t* and Mann–Whitney *U* tests to compare the continuous variables and the chi-square test to compare the categorical variables for each group. We used the Kaplan–Meier method to generate cumulative survival rates and compared them using the log-rank test to evaluate statistically significant differences. *P* < 0.05 was considered to indicate a statistically significant result. Statistical analysis was performed using SPSS version 22.0.

## 3. Results

### 3.1. Case Report

The patient is a 66-year-old man who underwent a right radical nephrectomy because of RCC in May 2019. The postoperative pathological diagnosis was clear cell renal cell carcinoma (ccRCC), Furhman grade 2, clinical stage T2aN0M0. No treatment was given after discharge, and no recurrence was found in CT follow-up six months after surgery. In February 2020, he began to notice melena. In October 2020, the patient was presented to our hospital complaining of recurrently intermittent melena for more than 8 months. Gastroscopy and pathology in another hospital revealed a chronic gastric ulcer without improvement after symptomatic treatment. He was diagnosed with cavernous hemangioma of the liver. Besides, no other disease in his past medical history and the physical examination was unremarkable. The gastroscopic biopsy was repeated in our hospital to determine the cause of melena. Gastric metastasis of RCC was confirmed by pathology.

**Figure 1:**
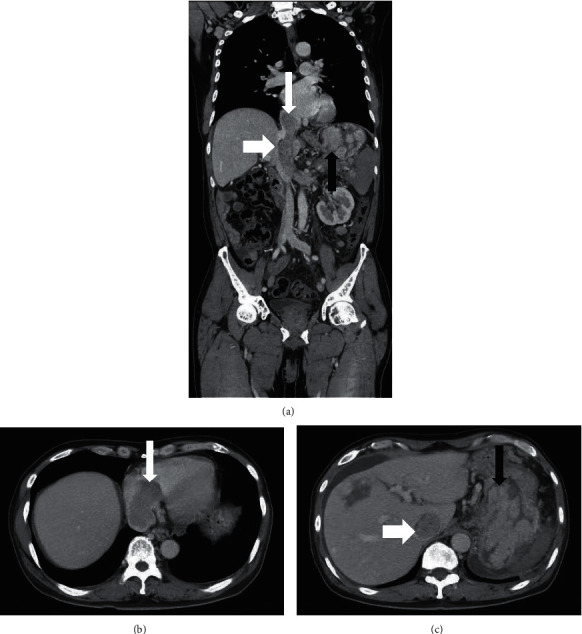
Enhanced CT of venous phase images of the patient. (a) CT enhanced coronal section with three-dimensional reconstruction. (b, c) CT enhanced transverse sections. The long white arrow in (a) and (b) indicates right atrium cancer thrombus, the short white arrow in (a) and (c) indicates cancer thrombus in the inferior vena cava, and the long black arrow in (a) and (c) indicates gastric metastasis.

Meanwhile, enhanced CT of the thoracic, full abdomen, and pelvic cavity revealed heterogeneous enhancement of soft tissue density obstructed in the right atrium, inferior vena cava to the superior mesenteric vein, and splenic vein junction, and presented with filling defects in the venous phase, suggesting cancer thrombi (Figure 1). After a multidisciplinary consultation, pazopanib-targeted therapy was first given in consideration of the high risk of surgery. After 6-month targeted therapy, the patient returned to our hospital for reevaluation. The routine blood test indicated microcytic hypochromic anemia with RBC 3.67 × 10^12^/L, hemoglobin of 90 g/L, hematocrit 30.3%, MCH24.5pg, MCHC 97.0 g/L, and MCV 78 fL. Another remarkable blood test was albumin 30.2 g/L.

**Figure 2:**
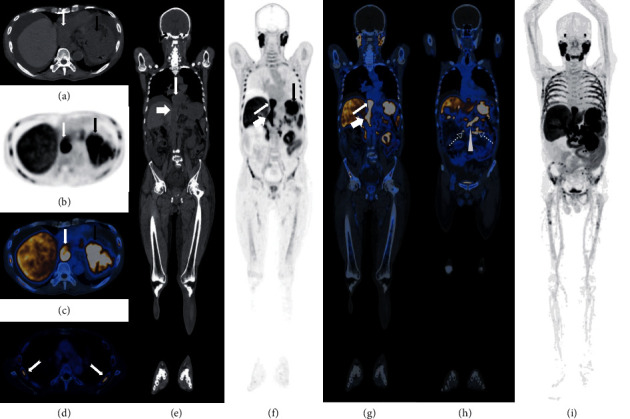
^18^F-PSMA PET/CT images of the same patient. (a–d) Transverse sections of CT, PET, and PET/CT fusion soft tissue window and PET/CT fusion bone window. (e–h) Coronal sections of CT, PET, and PET/CT fusion images, respectively. (i) PET MIP image. The long black arrow in (a–c) and (e–h) indicates gastric metastasis. The long white arrow in (a–c) and (e–g) indicates cancer thrombus in the right atrium. The short white arrow in (e-g) indicates cancer thrombus in the inferior vena cava. The white triangle in (h) indicates cancer thrombus in the splenic vein. The white dovetail arrows in (d) indicate multiple bone metastases. The white virtual arrows in (h) indicate abdominal lymph nodes.

At the same time, a whole-body^18^F-PSMA PET/CT scan was performed to evaluate the disease status and therapeutic efficacy (Figure 2): there was a large soft tissue mass in the gastric fundus (7.9 × 15.5 cm in size) with increased tracer uptake, and the maximum standardized uptake value (SUV_max_) was 16.2. Metastasis was considered based on his medical history. The increased tracer uptake in the right atrium, inferior vena cava, and splenic vein (SUV_max_14.8) was considered as cancer thrombi. Increased tracer uptake in multiple bones (SUV_max_9.9) and abdominal lymph nodes (SUV_max_22.0) were considered as metastases. In addition, the patient had lower extremity edema secondary to cancer thrombi.

**Figure 3:**
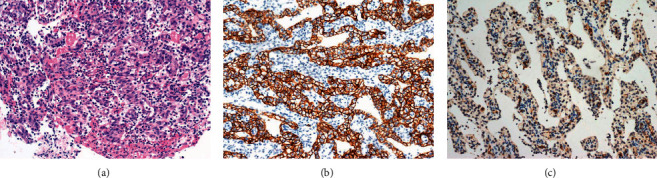
Microscopic biopsy and immunohistochemical images of the patient. (a) H&E stain revealed many small vascular hyperplasia and bright cytoplasmic tissue-like cells under the background of tissue inflammation and necrosis. (b, c) Immunohistochemistry stain revealed positive Carbonic Anhydrase IX (CAIX) and prostate-specific membrane antigen (PSMA).

Disease progression was assessed after ^18^F-PSMA PET/CT scanning, and the patient was subsequently treated with the combination of axitinib and tislelizumab. During the follow-up, the symptoms of gastrointestinal bleeding (melena) disappeared, and the disease was assessed as stable for 5 months, the time when we collected his medical data (Figure 3: microscopic biopsy and immunohistochemical images of the patient).

### 3.2. Literature Review

We identified 73 cases of RCC with gastric metastasis from literature reviews. Including the patient treated in our hospital, there were 5 cases of RCC with simultaneous solitary gastric metastasis [4, 16–19] (Table 1), 4 cases with gastric and multiple-site metastases [20–23] (Table 2), 21 cases with solitary metachronous gastric metastasis [16, 24–42] (Table 3), and 44 cases with metachronous gastric and multiple-site metastases [32, 43–76] (Table 4).

#### 3.2.1. Clinical Characteristics

The clinical characteristics of all cases are shown in Tables 1to 4. The median age of patients was 67 (from 48 to 87) years, with a majority occurred in male patients (53/73, 72.6%). The type of RCC in gastric metastasis was mostly ccRCC, with a proportion of 77.3% (57/74). The most gastrointestinal symptoms were upper gastrointestinal bleeding (melena or hematemesis) and anemia. As for the location of gastric metastasis, 42 cases had lesions in the body of the stomach, 22 in the fundus, and 11 in the pylorus, and the location of 2 cases involved two parts of the stomach. The median size of the lesions was 2.5 (from 0.3 to 15.5) cm. The appearance of lesions was polypoid or mass-like in 51cases (51/74, 68.9%) and ulceration in 8 cases (8/74, 10.8%); 15 cases (15/74, 20.3%) were not described in the literature. At the time of diagnosis of gastric metastasis, 48 cases had additional distal metastases; the main sites of metastasis were lung (34/48, 70.80%) and bone (14/48, 29.17%).

The modalities of examination for the diagnosis of gastric metastasis included upper gastrointestinal endoscopy (UGIE) (54 cases), CT (32 cases), endoscopic ultrasonography (EU) (7 cases), bone scintigraphy (5 cases), and PET/CT (4 cases). Among the 67 patients reported for treatment in the literature, there were 27 (27/67, 40.3%) patients who received surgical treatment, 15 (15/67, 22.39%) endoscopic treatment, 14 (14/67, 21.90%) drug treatment (including six targeted treatments), and 11 (11/67, 16.42%) palliative treatment. An overall median interval time between nephrectomy and the diagnosis of gastric metastasis was 6 (from 0.1 to 23) years; solitary metachronous gastric metastasis was 7 (from 1 to 23) years; metachronous gastric and multiple-site metastases was 5 (from 0.1 to 15) years.

#### 3.2.2. Comparison of Clinical Characteristics in Different Metastasis Models

Table 5 compares the clinical characteristics between solitary and multiple-site metastases and synchronous and metachronous gastric metastasis from RCC. The time of solitary gastric metastasis was longer than gastric metastasis with multiple-site metastases (7 vs. 5 years; *P*=0.034). The therapy choices were different statistically between solitary and multiple-site metastases (*P*=0.047), and patients with multiple-site metastases were more likely to receive drug and palliative treatment. For patient outcomes, gastric metastasis with multiple-site metastases had higher mortality than patients with solitary metastasis (17 vs. 1; *P*=0.028). There were no significant differences in age, gender, or characteristics of gastric metastasis (location, size, number, and shape) between solitary metastasis and multiple-site metastases. There were no significant differences in the clinical characteristics between synchronous and metachronous metastasis.

#### 3.2.3. Comparison of Clinical Characteristics in Survival

Table 6 compares the clinical characteristics affecting survival in gastric metastasis from RCC. The overall median patient survival after treatment was 14 (from 0.25 to 72) months in our systematic review. Specifically, solitary gastric metastasis was 12 (from 2 to 58) months, gastric and multiple-site metastases was 24 (from 0.25 to 72) months, synchronous gastric metastasis was 6 (from 2 to 12) months, and metachronous gastric metastasis was 17 (from 0.25 to 72) months. The patients with synchronous gastric metastasis had a shorter survival time than metachronous gastric metastasis (6 vs. 17 months; *P*=0.018). There were no significant differences in other clinical characteristics.

## 4. Discussion

Clinically, RCC most commonly occurs in the age 60–70 years old, with a higher incidence rate in men than women (about 2 : 1) [77]. RCC tends to spread in different ways, such as lymphatic, hematogenous, direct invasion, et al. The most common metastatic sites of RCC are lung and bone [78], and gastric metastasis is rare. In the current study, we reported a rare case of RCC with gastric metastasis and multiple other metastases after radical RCC surgery, and metastases lesions were avid to PSMA in the ^18^F-PSMA PET/CT imaging.

The diagnosis of RCC with gastric metastasis was mostly upper gastrointestinal endoscopy (UGIE) and CT. Other tests included endoscopic ultrasonography (EU), bone scintigraphy, and PET/CT, which was used to accurately stage the disease. Gastrointestinal endoscopy plays an important role in the diagnosis of gastrointestinal diseases. It can display mucosal and intraluminal lesions and be used for biopsy sampling and treatment. However, it is limited in showing early submucosal and extra-luminal lesions and is not the preferred examination for follow-up patients in clinical practice. By contrast, CT is a common practice in the follow-up of malignant diseases; particularly, enhanced CT is of great help for the diagnosis and differentiation of diseases. For example, in this case, the cancer thrombus showed as an irregular enhancement of soft tissue mass on enhanced CT and filling defect in the venous phase, which is helpful for the differentiation of vascular thrombus (vascular thrombus does not visible on enhanced CT). For the primary tumor, due to the lack of specific tumor markers, pretreatment diagnosis and posttreatment follow-up of RCC were based on imaging, including upper abdominal enhanced CT, upper abdominal magnetic resonance imaging (MRI), and PET/CT. CT is of help in the pretreatment evaluation of RCC, including the determination of stage, vascular invasion, renal pelvis, ureter invasion, et al., which can guide the surgical method and resection scope. Although CT is currently recommended by the European Association of Urology (EAU) as a routine follow-up monitoring method after RCC treatment [79], it is difficult to find small lesions due to its low sensitivity. CT has limitations in distinguishing postoperative changes from recurrence and finding early or whole-body metastatic lesions. As mentioned earlier, RCC has the potential for multiple-site metastases.

Unlike traditional morphological imaging technology, the molecular imaging agent of PSMA PET/CT can specifically target the lesion with the expression of PSMA, which can reveal the characteristics of lesions at the molecular level with high sensitivity. In prostate cancer screening, PSMA PET/CT has been written into the guidelines for pre- and posttreatment screening in patients with local and systemic metastatic prostate cancer to assess treatment response [80]. The application of PSMA PET/CT in RCC has been attracting attention in recent years. A study [81] has proposed that PSMA PET/CT can be used as an imaging method for staging and restaging of RCC to improve the staging sensitivity, which can directly lead to the change of treatment in some cases, particularly, in the application of metastatic ccRCC. Gao et al. [82] retrospectively examined the data of 36 ccRCC patients with preoperative ^68^Ga-PSMA-11 PET/CT scan parameters (including maximal tumor diameter, mean CT value, and SUV_max_) and surgical specimens (including WHO/ISUP grade and adverse pathology). They found that SUV_max_ could effectively differentiate WHO/ISUP grade (3-4 vs. 1-2) and adverse pathology (positive vs. negative) (both *P* < 0.001), however, with no difference in CT value, suggesting that PSMA PET/CT molecular imaging can reflect the pathological features of ccRCC. In addition, some studies found that PSMA PET/CT can provide a more accurate response assessment after the treatment of metastatic RCC [7], expecting it to become a valuable tool for posttreatment evaluation [83]. For example, Siva et al. [83] investigated the differential role of ^18^F-fluorodeoxyglucose (FDG) and PSMA-PET/CT scanning in patients with oligometastatic RCC, in particular the utility of PSMA PET/CT for assessment of diagnosis and therapeutic response. PSMA uptake was typically more intense than FDG in RCC, which was particularly important in oligometastatic disease, where improved diagnostic sensitivity may impact management decisions. More importantly, both modalities demonstrated response earlier than morphological appearance on CT or MRI imaging. Therefore, PSMA could become a promising tool in diagnostic and therapeutic response assessment in patients with metastatic RCC. To the best of our knowledge, there have been no reports of RCC diagnosis with gastric metastasis by PSMA PET/CT. In this case, ^18^F-PSMA PET/CT revealed multiple metastases after treatment, providing an important reference for evaluating therapeutic efficacy and treatment guidance.

In our systematic review, we found that the majority of gastric metastasis occurred in male patients, with common symptoms of upper gastrointestinal bleeding and anemia. The lesions of gastric metastasis were mainly single, small, polypoid, or mass-like and located in the middle part of the stomach. By contrast, primary gastric cancer mostly originates from the gastric mucosa, which is more common in the lower part of the stomach, such as the gastric antrum and gastric lesser curvature. The lesion is mainly ulcer type and often accompanied by chronic gastritis, gastric ulcer, or Helicobacter pylori infection. The combination of medical history and imaging findings is helpful for the diagnosis and differential diagnosis of the disease, but the final diagnosis still depends on biopsy.

At the time of diagnosis of RCC with gastric metastasis, most patients presented multiple metastatic sites, mainly lung and bone; other rare metastatic sites included thyroid, skin, testes, and bladder. The case in our report was accompanied by cancer thrombi in the right atrium, inferior vena cava, and splenic vein, without lung metastasis, which was not previously reported. In cancer metastasis, the appearance of cancer thrombus is formed through direct invasion or blood spreading, mostly located in the renal vein or lower vena cava, according to the blood flow pathway. In this case, the presence of distant and multiple cancer thrombi but spare of renal vein or lower vena cava metastasis was rare. The mechanism of RCC with gastric metastasis is currently unclear. However, it is worth noting that the type of RCC in gastric metastasis was mainly ccRCC. Clear cell RCC has a rich blood supply compared to other types of RCC and can be transferred to other organs in various ways [20, 84]. This feature may be responsible for gastric metastasis. Thus, the presence of this morphology in any unknown gastric lesion should direct further investigations to exclude metastatic RCC, even in the absence of such a prior diagnosis.

In our analysis, the overall median interval time from nephrectomy to the diagnosis of gastric metastasis was 6 years, and the overall median survival was 14 months. We noticed that the modes of gastric metastasis were related to survival and prognosis. Compared with the solitary gastric metastasis of RCC, gastric metastasis with multiple-site metastases had a relatively shorter interval time and higher mortality during the follow-up. Compared with metachronous gastric metastasis, patients with synchronous gastric metastasis had shorter median survival, suggesting a poor prognosis. Our data were in consistent with that reported in several other studies [21, 24, 51, 85, 86] from different countries where it was reported that the median interval range was 6.3 to 8.5 years and the median survival was 19 months. It can be seen that the time to gastric metastasis of RCC was long after treatment of the primary tumor. In addition, even if metachronous gastric metastasis appeared, the prognosis was better than synchronous gastric metastasis after treatment. Therefore, regular follow-up is important.

The optimal treatment for RCC patients with gastric metastasis remains unclear, and the treatment measures taken are different according to the status of gastric metastasis. Endoscopic or surgical treatment can be used for single and early gastric metastasis [24]. Immunotherapy and/or targeted therapy can be used for multiple focal metastases or other organ metastases to improve patient survival [87–90]. The treatment of cancer thrombus requires a comprehensive evaluation and individualized therapy, including surgical resection, biological regulators, or targeted molecular therapy [87, 91, 92]. Although there was no statistical difference in the survival rates in our statistics, patients with treatment had a better survival rate than those untreated. The patient, in this case, received targeted and immunotherapy for nearly 1 year, and the evaluation result is good with a stable state at present.

## 5. Conclusion

RCC with gastric metastasis is rare. Postoperative follow-up with multiple imaging modalities for monitoring recurrence and metastasis is necessary and important. The application of PSMA PET/CT in RCC monitoring, especially in metastatic ccRCC, has attracted increasing attention. PSMA PET/CT is expected to provide a powerful tool for disease staging, restaging, and evaluation of treatment efficacy.

## Figures and Tables

**Table 1:** Summary of RCC cases with solitary synchronous gastric metastasis.

Case	Author	Year	Age (years)	Gender	Type	Symptoms	Location	Size (cm)	Number	Shape	Treatment	Examination	Outcome
1	Sukumaran et al. [18]	2012	63	M	ccRCC	Dysphagia, weight loss	U	4	1	Mass	Esophagogastroctomy	ND	ND
2	Greenwald et al. [19]	2014	62	M	ccRCC	Asymptomatic	U	3	1	Mass	Partial gastrectomy	UGIE, CT	8-month SAT
3	Weissman et al. [16]	2019	70	M	RCC	Anemia, malaise, weight loss	U	1	1	Polypoid	Palliative treatment	UGIE	ND
4	Koterazawa et al. [4]	2021	70	F	ccRCC	Weight loss	M	0.3	1	ND	Endoscopic treatment	UGIE	4-month SAT
5	Parmar et al. et al. [17]	2021	64	M	ccRCC	Hematemesis, weight loss	U	ND	1	Mass	Partial gastrectomy	UGIE, CT	12-month SAT

U, upper part of the stomach; M, middle part of the stomach; L, lower part of the stomach; UGIE, upper gastrointestinal endoscopy; SAT, survival after therapy; ND, not described.

**Table 2:** Summary of RCC cases with synchronous gastric and multiple-site metastases.

Case	Author	Year	Age (yeas)	Gender	Type	Symptoms	Location	Size (cm)	Number	Shape	Treatment	Additional metastases	Examination	Outcome
6	Kibria et al. [23]	2009	53	M	ccRCC	Melena, fatigue, dizziness	U	1.5	1	Polypoid	Palliative treatment	Lung, bone	UGIE, CT	Died 2-month AT
7	Tiwari et al. [22]	2010	58	F	ccRCC	Melena, hematemesis, fatigue, dizziness	L	4	1	Mass	Subtotal gastrectomy	Lung	UGIE, CT	Died 2-month AT
8	Kim et al. [21]	2012	79	M	ccRCC	Abdominal pain	M	0.6	1	Ulcerated	Endoscopic treatment	Lung, bone	UGIE, CT, PET/CT	6-month SAT
9	Arakawa et al. [20]	2018	80	F	ccRCC	Anorexia	M	1	1	Ulcerated	Target treatment	Lung, liver	UGIE, EU, CT	ND

U, upper part of the stomach; M, middle part of the stomach; L, lower part of the stomach; UGIE, upper gastrointestinal endoscopy; EU, endoscopic ultrasonography; SAT, survival after therapy; AT, after therapy; ND, not described.

**Table 3:** Summary of RCC cases with solitary metachronous gastric metastasis.

Characteristics	*N* (%)
Median age (range) (years)	69 (50–85)
Gender	
Female	15 (71.4)
Male	5 (23.8)
ND	1 (4.8)
Type	
ccRCC	17 (81.0)
RCC	4 (19.0)
Symptoms	
UGIB (melena or hematemesis)	9 (42.9)
Anemia	9 (42.9)
Others	2 (9.5)
Asymptomatic	2 (9.5)
ND	4 (19.0)
Primary location (stomach)	
Upper	8 (38.1)
Middle	10 (47.6)
Lower	3 (14.3)
Median size (range) (cm)	3 (0.6–10.0)
Numbers of the primary diseases	
1	20 (95.2)
2	1 (4.8)
Shape of the primary diseases	
Polypoid/mass	18 (85.7)
Ulcerated	2 (9.5)
ND	1 (4.8)
Treatment	
Surgery	9 (42.9)
Endoscopy	6 (28.6)
Drug	2 (9.5)
ND	4 (19.0)
Examination	
UGIE	18 (85.7)
CT	8 (38.1)
EU	3 (14.3)
Bone scintigraphy	2 (9.5)
BCR	2 (9.5)
PET/CT	1 (4.8)
MRI	1 (4.8)
Median interval time (years)	7 (1–23)
Follow-up outcomes	
Survival after therapy	9 (42.9)
Died after therapy	1 (4.8)
ND	11 (52.4)
Median survival (months)	15 (2–58)

UGIB, upper gastrointestinal bleeding; UGIE, upper gastrointestinal endoscopy; EU, endoscopic ultrasonography; BCR, barium contrast radiography; ND, not described.

**Table 4:** Summary of RCC cases with metachronous gastric and multiple-site metastases.

Characteristics	*N* (%)
Median age (range) (years)	67 (48–87)
Gender	
Female	32 (72.7)
Male	12 (27.3)
Type	
ccRCC	32 (72.7)
RCC	8 (18.2)
ND	4 (9.1)
Symptoms	
UGIB (melena or hematemesis)	23 (52.3)
Anemia	14 (31.8)
Others	6 (13.6)
Asymptomatic	2 (4.5)
ND	5 (11.4)
Primary location (stomach)^*∗*^	
Upper	9 (19.6)
Middle	29 (63.0)
Lower	7 (15.2)
ND	1 (2.2)
Median size (range) (cm)	2.5 (0.4–15.5)
Numbers	
1	29 (65.9)
2	3 (6.8)
Multiple	6 (13.6)
ND	6 (13.6)
Shape of the primary diseases	
Polypoid/mass	27 (61.4)
Ulcerated	4 (9.1)
ND	13 (29.5)
Treatment	
Surgery	14 (31.8)
Endoscopy	7 (15.9)
Drug	11 (25.0)
Palliative	9 (20.5)
ND	3 (6.8)
Additional metastases	
Lung	30 (68.2)
Bone	11 (25.0)
Pancreas	10 (22.7)
Liver	7 (15.9)
Brain	7 (15.9)
Adrenal	4 (9.1)
Pleura	2 (4.5)
Others (thyroid, gallbladder, rectum, testes, bladder, skin, and cancer thrombus)	7 (each one) (15.9)
Examination	
UGIE	29 (65.9)
CT	18 (40.9)
EU	3 (6.8)
Bone scintigraphy	3 (6.8)
BCR	1 (2.3)
PET/CT	2 (4.6)
Median interval time (years)	5 (2–8.5)
Follow-up outcomes	
Survival after therapy	18 (40.9)
Died after therapy	15 (34.1)
ND	11 (25.0)
Median survival (months)	24 (0.25, 72)

UGIB, upper gastrointestinal bleeding; UGIE, upper gastrointestinal endoscopy; EU, endoscopic ultrasonography; BCR, barium contrast radiography; ND, not described; ^*∗*^location of 2 cases involved two parts of the stomach.

**Table 5:** Comparison of clinical characteristics between solitary gastric metastasis and multiple-site metastases and synchronous and metachronous gastric metastasis from RCC.

Characteristics	Solitary metastasis and multiple-site metastases		Synchronous metastasis and metachronous metastasis	
	Statistics	*P* value	Statistics	*P* value
Median age (years)	0.743	0.460	−0.546	0.585
Gender	0.221	0.639	0.001	0.978
Location (U, M, and L)	6.009	0.050	3.656	0.161
Median tumor size (cm)	0.023	0.982	−1.374	0.169
Shape (polypoid/mass and ulcerated)	0.943	0.332	0.213	0.645
Median interval time (years)	−2.116	0.034^a^		—
Therapy (surgery, endoscopy, drug, and palliative)	7.948	0.047^a^	0.734	0.865
Outcomes (survival and death)	4.834	0.028^a^	0.000	1.000

U, upper part of the stomach; M, middle part of the stomach; L, lower part of the stomach; ^a^ difference is statistically significant.

**Table 6:** Comparison of clinical characteristics affecting survival in gastric metastasis from RCC.

Characteristics	Statistics	*P* value
Median age (67 years)	0.845	0.358
Gender	0.692	0.405
Location (U, M, and L)	1.981	0.576
Median tumor size (2.5 cm)	0.483	0.487
Shape (polypoid/mass and ulcerated)	2.211	0.137
Median interval time (6 years)	0.440	0.507
Therapy (surgery, endoscopy, drug, and palliative)	1.283	0.733
Metastasis (solitary and multiple-site metastases)	2.077	0.150
Occurrence time (synchronous and metachronous)	5.605	0.018^a^

U, upper part of the stomach; M, middle part of the stomach; L, lower part of the stomach; ^a^ difference is statistically significant.

## Data Availability

Data used to support the findings of this study are available from the corresponding author upon request.
